# Gastroprotective Effect of Enteral Nutrition Formula in Mice Injected Subcutaneously with Indomethacin

**DOI:** 10.3390/nu13093297

**Published:** 2021-09-21

**Authors:** Yoshiaki Yamagishi, Rei Saiki, Takeshi Yoshimi, Toshiyuki Kudo, Kiyomi Ito

**Affiliations:** 1 Research Institute of Pharmaceutical Sciences, Musashino University, 1-1-20 Shinmachi, Nishitokyo-shi, Tokyo 202-8585, Japan; y_yama@musashino-u.ac.jp (Y.Y.); s1543108@stu.musashino-u.ac.jp (R.S.); t.yoshimi68@gmail.com (T.Y.); to_kudou@musashino-u.ac.jp (T.K.); 2 Department of Pharmacy, Ogikubo Hospital, 3-1-24 Imagawa, Suginami-ku, Tokyo 167-0035, Japan

**Keywords:** indomethacin, subcutaneous injection, nonsteroidal anti-inflammatory drugs, enteral nutrition formula, gastric lesions

## Abstract

We have previously shown that two enteral nutrition formulas suppressed gastric lesions induced by the oral administration of indomethacin (IND) in mice. However, the mechanism of their protective effect is unknown. In this study, the effect of the two enteral nutrition formulas on gastric lesions induced by subcutaneous IND injection was investigated, with the objective of exploring the possibility that they may interact directly with IND in the gastrointestinal tract. Ten-week-old, male, ICR mice were fasted, then orally given either purified water, Mermed^®^ One, or 2-fold diluted Terumeal^®^ 2.0α as enteral nutrition formula (25 mL/kg). IND was injected subcutaneously at 20 mg/kg after 30 min, and the stomach was removed 6 h later and fixed in formalin. The number and area of lesions in the stomachs of mice given enteral nutrition formula was reduced to 56–89% and 34–61%, respectively, compared with the mice given purified water. The time courses of plasma IND concentrations were comparable among the three groups. These results suggested that the effect of these enteral nutrition formulas on gastric lesions did not originate from their direct interaction with IND in the gastrointestinal tract or their effect on the disposition of IND.

## 1. Introduction

Nonsteroidal anti-inflammatory drugs (NSAIDs) are widely used in clinical practice; for example, to suppress inflammation in orthopedic surgical disorders and to inhibit platelet aggregation in coronary artery disease [1,2]. NSAID-induced ulcers are a side effect of NSAIDs that cannot be ignored. Proton pump inhibitors (PPIs) and prostaglandin (PG) analogs are often used to treat NSAID-induced ulcers [3], but their side effects [4,5,6,7,8,9] and drug interactions with other medications [10,11,12,13,14] are a concern.

We have been investigating enteral nutrition formulas with the aim of resolving this issue, and we have previously shown that oral ingestion of two of these (Mermed^®^ One and Terumeal^®^ 2.0α, Terumo Corporation, Tokyo, Japan, which are classified as foods) act to suppress gastric lesions induced by the oral administration of indomethacin (IND) in mice. Neither of these two formulas affected the blood concentration of IND when it was administered orally [15]. These results suggested that these two enteral nutrition formulas may provide an alternative to drugs for the treatment of NSAID-induced ulcers, and, as such, they may be one means of resolving the problem of polypharmacy [16].

However, if the effect of these enteral nutrition formulas on gastric lesions is the result of direct interactions with IND present at the same time in the gastrointestinal tract, they may only be effective in preventing gastric lesions induced by oral NSAIDs. In addition to oral formulations, several other administration routes are also used for NSAIDs, including injections [17,18,19,20] and suppositories [21,22]. Intravenous injection of IND [17], ibuprofen [18], or flurbiprofen [19], and intramuscular injection of ketoprofen [20] have been reported to cause gastric lesions, although the incidence is unknown. The incidence of gastric ulcers due to the use of IND suppositories has been shown to be the same as that for its oral use [23].

In this study, the effect of these enteral nutrition formulas in suppressing gastric lesions induced by subcutaneous IND injection in mice was investigated, with the objective of confirming whether their effect was due to their direct interaction with IND in the gastrointestinal tract.

## 2. Materials and Methods

### 2.1. Reagents

The enteral nutrition formulas used were Mermed^®^ One (400 kcal/400 mL) and Terumeal^®^ 2.0α (400 kcal/200 mL). Each was purchased from Terumo Corporation (Tokyo, Japan), and their ingredients are listed in the supplementary table of our previous report [15]. The IND was purchased from Nacalai Tesque (Kyoto, Japan). The carboxymethyl cellulose (CMC), isoflurane, and 10% neutral-buffered formalin were purchased from FUJIFILM Wako Pure Chemical Corporation (Tokyo, Japan). The IND-*d*_4_ was purchased from Cayman Chemical (Ann Arbor, MI, USA). All other reagents used were of the highest commercially available grade.

### 2.2. Animals

Male ICR mice aged 10 weeks (Sankyo Labo Service Corporation, Tokyo, Japan) were used. The temperature (23.3 ± 0.1 °C) and humidity (54.8 ± 1.1%) were maintained in an air-conditioned room, and animals were housed in suitable cages with a 12 h light–dark cycle (lights on from 08:00 to 20:00) with free access to food and water unless otherwise stated. All animal experiments were conducted with the approval of the Institutional Animal Care and Use Committee of Musashino University.

### 2.3. Dosing and Blood Sampling Schedule

**Figure 1:**
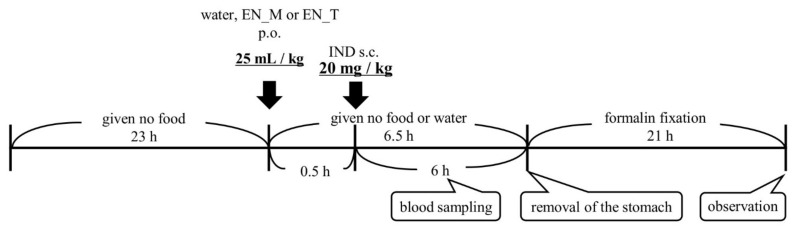
Experimental protocol. IND: indomethacin; EN_M: Mermed^®^ One; EN_T: Terumeal^®^2.0α diluted by a factor of 2 with purified water to reduce the caloric content to that of EN_M; p.o.: per os; s.c.: subcutaneous.

Mice were fasted for 23 h, administered either purified water or an enteral nutrition formula (25 mL/kg, orally), and then given IND (20 mg/kg, subcutaneously) 30 min later (*n* = 5 per group). The animals were given no food or water throughout up to removal of the stomach under isoflurane anesthesia 6 h later. Mermed^®^ One (EN_M) and Terumeal^®^ 2.0α diluted by a factor of 2 with purified water to reduce the caloric content to that of EN_M (EN_T) were used as the enteral nutrition formulas. The IND was administered as a 1% CMC suspension (2 mg/mL) (Figure 1). An aliquot of 25–30 µL of blood was collected from the tail vein in a heparinized capillary tube (Hirschmann, Eberstadt, Germany) 0.25, 0.5, 1, 2, 4, and 6 h after IND administration. Just after collection, blood was centrifuged for 5 min at 14,800× *g* (H-1200F, Kokusan, Saitama, Japan), and the resulting plasma was stored at −80 °C.

### 2.4. Observation of Gastric Lesions Induced by IND

The pyloric region of each removed stomach was ligated, and the stomach was filled by injecting 2 mL of 10% neutral-buffered formalin into the cardiac region. Following fixation of the mucosal surface for 21 h, an incision was made along the greater curvature to open the stomach. A magnifying glass with 7× magnification (Peak Scale Lupe 7×, Tohkai Sangyo, Tokyo, Japan) was used to determine the number and area (in square millimeters) of gastric lesion sites. The photographs were taken with a camera by placing the mucosa between transparent plates on a luminescent device (TREVIEWER^®^ A4-500, Trytec, Kyoto, Japan).

### 2.5. Analysis of Plasma IND Concentrations

First, 90 µL of acetonitrile (containing 2.5 µM IND-*d*_4_ as the internal standard) was added to 10 µL of each plasma sample, and the resulting mixture was vortexed for 30 s. Samples were then centrifuged (4 °C, 8,000× *g*, 10 min) using an Eppendorf 5427R centrifuge (Eppendorf, Hamburg, Germany). The IND concentration in the supernatant was determined with a liquid chromatography–tandem mass spectrometry (LC-MS/MS) instrument (LCMS-8040; Shimadzu, Tokyo, Japan). The column was a GL Sciences Inertsil ODS-3 (particle size: 5 µm; inside diameter: 4.6 mm; length: 150 mm; GL Sciences, Tokyo, Japan), the mobile phase was a 3:7 mixture of purified water (0.1% formic acid) and acetonitrile (0.1% formic acid), the column temperature was 40 °C, the flow rate was 0.5 mL/min, and ionization conditions were electrospray ionization-positive, with *m/z* 358.00 > 139.05 IND and *m/z* 362.10 > 142.85 IND-*d*_4_ (internal standard) detected as ions.

### 2.6. Calculation of Pharmacokinetic Parameters

The trapezoidal rule was used to calculate the area under the plasma concentration-time curve to 6 h postdose (AUC_0–6_) from time courses of plasma IND concentrations. Maximum plasma concentration (C_max_) and time to C_max_ (T_max_) were determined from time courses of plasma IND concentrations. Mean residence time (MRT) was calculated by dividing the area under the first moment curve to 6 h postdose (AUMC_0–6_) as determined with the trapezoidal rule by AUC_0–6_.

### 2.7. Statistical Analysis

All data are expressed as mean±standard deviation (S.D.). Tukey’s test was performed using SPSS software (version 24; IBM, Tokyo, Japan) to test for significant differences. Values less than the critical rate of 5% (*p* < 0.05) were considered statistically significant.

## 3. Results

### 3.1. Effect of Enteral Nutrition Formulas on Gastric Lesions Induced by Subcutaneous IND Injection

**Figure 2:**
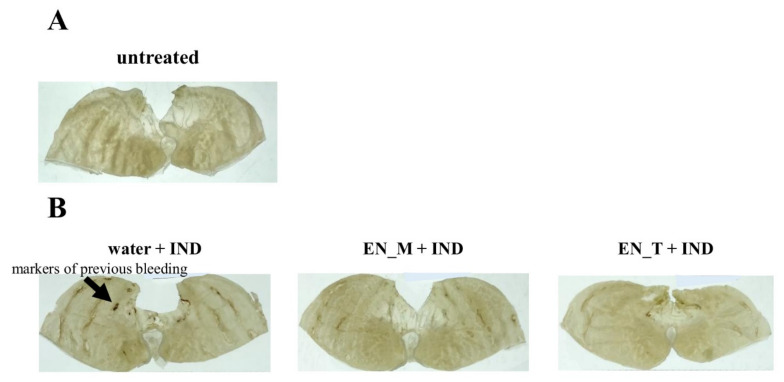
Effects of EN_M and EN_T on IND-induced gastric lesions. Figures show representative photographs of stomachs taken after fixation for 21 h in 10% neutral-buffered formalin following removal 6 h after subcutaneous IND (20 mg/kg) administration. (**A**) Untreated group; (**B**) group treated with 25 mL/kg of purified water, EN_M, or EN_T orally 30 min before subcutaneous IND administration.

No obvious gastric lesions were observed in the untreated group (Figure 2A). In the mice injected with IND, gastric lesions were present, with marks of previous bleeding clearly evident (Figure 2B). Of the mice injected with IND, gastric lesions tended to be suppressed in the EN_M + IND and the EN_T + IND groups compared with the water + IND group (Figure 2B).

**Figure 3:**
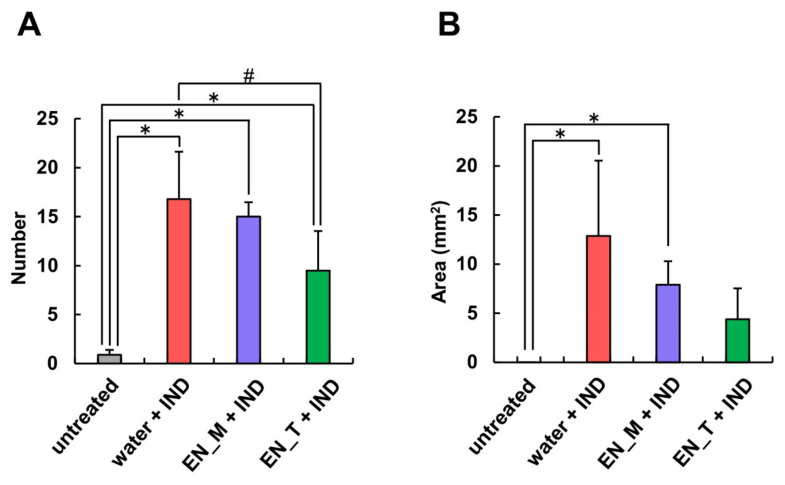
Effects of EN_M and EN_T on numbers and areas of gastric lesions induced by IND. Gross observations were performed to determine numbers (**A**) and areas (**B**) of lesions in stomachs taken after fixation for 21 h in 10% neutral-buffered formalin following removal 6 h after subcutaneous IND (20 mg/kg) administration. The gray column represents the untreated group; the red column, blue column, and green column represent the group treated with purified water, EN_M, and EN_T, respectively, 30 min before subcutaneous IND administration. Each column and vertical bar represents mean + S.D., *n* = 5. * *p* < 0.05 vs. untreated group and # *p* < 0.05 vs. water + IND group (Tukey’s test).

The number and area of gastric lesions were both greatest in the water + IND group, with fewer gastric lesions present in both the EN_M + IND and the EN_T + IND groups (Figure 3). Compared with the untreated group, the number of gastric lesions was significantly higher in all other groups. Compared with the water + IND group, the number of lesions was 89% in the EN_M + IND group and 56% in the EN_T + IND group, a significant decrease in the case of the EN_T + IND group (Figure 3A). The area of gastric lesions was significantly greater in the water + IND group and the EN_M + IND group compared with that in the untreated group. The area in the EN_M + IND group was 61% of that in the water + IND group, and that in the EN_T + IND group was 34% of that in the water + IND group (Figure 3B).

### 3.2. Effect of Enteral Nutrition Formulas on Plasma IND Concentration

**Figure 4:**
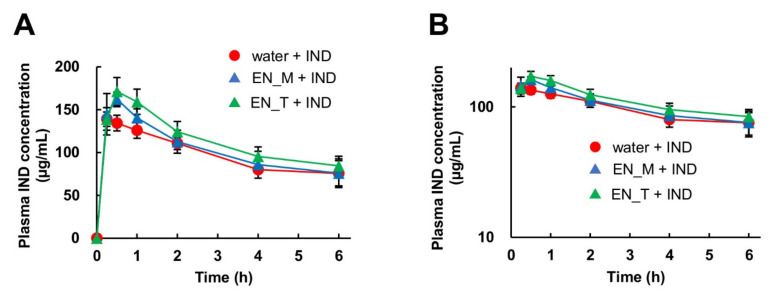
Effects of EN_M and EN_T on plasma IND concentrations. Blood was collected from the mice orally given 25 mL/kg of purified water, EN_M, or EN_T 30 min before subcutaneous IND (20 mg/kg) administration. LC-MS/MS was used to determine plasma IND concentrations. (**A**) Normal plot; (**B**) semilog plot. The closed red circles, blue triangles, and green triangles represent the profiles for the group treated with purified water, EN_M, and EN_T, respectively, orally 30 min before subcutaneous IND administration. Each symbol represents mean ± S.D., *n* = 5.

Although the plasma IND concentration tended to be slightly higher in the EN_M + IND and EN_T + IND groups compared with the water + IND group, the plasma IND concentrations in all groups were comparable (Figure 4).

The AUC_0–6_ for IND was 1.1-fold higher in the EN_M + IND group and 1.3-fold (significantly) higher in the EN_T + IND group compared with the water + IND group. The C_max_ was 1.1-fold higher in the EN_M + IND group and 1.2-fold higher in the EN_T + IND group compared with in the water + IND group, and this difference was significant for both groups. There were no significant differences between any of the groups in T_max_ or MRT (Table 1).

## 4. Discussion

We have previously shown that the oral administration of two types of enteral nutrition formula (EN_M and EN_T) was effective in suppressing gastric lesions induced by oral IND administration in mice [15]. It was suggested that the mechanism whereby orally administered enteral nutrition formulas prevented gastric lesions might involve some sort of interaction between the formula and IND while both were simultaneously present in the gastrointestinal tract. In the present study, therefore, the effectiveness of the above two enteral nutrition formulas in preventing gastric lesions induced by subcutaneous IND injection in mice was investigated.

When formalin-fixed gastric mucosa was observed through a loupe, no obvious gastric lesions were seen in the untreated group (Figure 2A). On the other hand, such lesions were present in the groups that had received subcutaneous IND injections (Figure 2), indicating that these injections had successfully produced a mouse model of gastric lesions. The results of these observations were similar to those seen when the same IND dose was administered orally [15]. Measurements of the number and area of gastric lesions showed that they were both lower in the EN_M + IND and EN_T + IND groups than in the water + IND group (percentage of numbers: EN_M + IND 89%, EN_T + IND 56%; area: EN_M + IND 61%, EN_T + IND 34% compared to the water + IND group) (Figure 3). Although the effects of these enteral nutrition formulas in inhibiting gastric lesions were weaker in comparison with their effects after the oral administration of IND (percentage of numbers: EN_M + IND 51%, EN_T + IND 60%; area: EN_M + IND 31%, EN_T + IND 24% compared to the water + IND group) [15], these results suggested that they were not the result of their direct interaction with IND in the gastrointestinal tract.

A comparison of the plasma IND concentrations found that, in the water + IND group, the AUC_0–6_ and C_max_ of IND after subcutaneous injection were approximately two times higher than those after oral administration of the same dose of IND, possibly reflecting first-pass metabolism after oral administration. As with oral IND administration, the AUC_0–6_ and C_max_ for IND following its subcutaneous injection increased in the order water + IND, EN_M + IND, and EN_T + IND (Table 1), suggesting that some of the ingredients in these two enteral nutrition formulas may affect the metabolism or excretion of IND, increasing its concentration in blood. However, even in the EN_T + IND group, the AUC_0–6_ increased by only 1.3-fold and the C_max_ increased by only 1.2-fold, indicating that these enteral nutrition formulas did not greatly affect the disposition of IND.

The present results demonstrated that the two enteral nutrition formulas used in this study inhibited gastric lesions irrespective of whether IND was administered orally or subcutaneously. They may therefore prevent gastric lesions induced by IND administered by routes other than oral administration. These results at least suggest that the gastroprotective effect exerted by these enteral nutrition formulas was not the result of their direct interaction with IND in the gastrointestinal tract, and that they did not affect the disposition of IND.

Although the mechanism of the gastroprotective effect of the enteral nutrition formulas is unknown, since NSAIDs cause gastric lesions by inhibiting cyclooxygenase (COX)-1, which is constitutively expressed in the gastric mucosa [24,25], it is possible that the enteral nutrition formulas used in this study alleviated the inhibition of COX-1 by IND. Further studies are needed to identify the detailed mechanism by which these two enteral nutrition formulas inhibit gastric lesions.

In our preliminary study, a glucose solution with the same calorie content as the enteral nutrition formulas were found to be ineffective for preventing IND-induced gastric lesions (data not shown), suggesting that the gastroprotective effects of the enteral nutrition formulas cannot be attributed solely to the calorie. Although sodium alginate has been reported to have a preventive effect on gastric lesions induced by IND in rats and mice [26,27], both of the two enteral nutrition formulas, with or without sodium alginate, showed a gastroprotective effect in our study. The content of sodium alginate in EN_M is unknown, but assuming that sodium alginate is the only dietary fiber (8.3 µg/mL) in EN_M, the dose of sodium alginate in the EN_M group is estimated to be approximately 275 mg/kg, which is lower than the doses reported to have gastroprotective effect (approximately 500 mg/kg [26] and 8000 mg/kg [27]). This might be the reason for the lack of difference in the effects between the two enteral nutrition formulas in the present study. It would be important to specify the effective component(s) of the enteral nutrition formulas in a future study. It will also be useful to explore the possibility that other enteral nutrition formulas may be similarly effective.

In order to demonstrate the further value of enteral nutrition formulas, it will also be necessary to explore their effects in inhibiting gastric lesions induced by NSAIDs other than IND, as well as other drug-induced gastric lesions caused by steroids [28,29], anticancer agents [30], bisphosphonates [31], coagulation-related drugs [32,33,34], and other medications. If the above studies were to show that enteral nutrition formulas can be used for prevention and treatment as alternatives to PPIs and other drugs, thus reducing the use of prescription medications, this might help improve the problems of drug interactions due to multiple medications, reduced drug adherence, and increased medical costs.

## 5. Conclusions

The effect of two enteral nutrition formulas (EN_M and EN_T) on IND-induced gastric lesions does not originate from their direct interaction with IND in the gastrointestinal tract or their effect on the disposition of IND.

## Figures and Tables

**Table 1:** Pharmacokinetic parameters calculated from time courses of plasma IND concentrations.

	Water + IND	EN_M + IND	EN_T + IND
T_max_ (h)	0.45 ± 0.29	0.45 ± 0.10	0.50 ± 0.00
C_max_ (µg/mL)	142 ± 12	163 ± 8 *	171 ± 16 *
AUC_0-6_ (µg·h/mL)	581 ± 23	614 ± 85	746 ± 19 *
MRT (h)	2.66 ± 0.09	2.61 ± 0.20	2.67 ± 0.02

Each value represents mean ± S.D., *n* = 5. * *p* < 0.05 vs water + IND (Tukey’s test). T_max_: time to C_max_; C_max_: maximum plasma concentration; AUC_0–6_: area under the plasma concentration-time curve to 6 h postdose; MRT: mean residence time.
